# Nurses’ spiritual attitudes and involvement—Validation of the Polish version of the Spiritual Attitude and Involvement List

**DOI:** 10.1371/journal.pone.0239068

**Published:** 2020-09-11

**Authors:** Alina Deluga, Beata Dobrowolska, Krzysztof Jurek, Barbara Ślusarska, Grzegorz Nowicki, Alvisa Palese

**Affiliations:** 1 Department of Family Medicine and Community Nursing, Faculty of Health Sciences, Medical University of Lublin, Lublin, Poland; 2 Department of Management in Nursing, Faculty of Health Sciences, Medical University of Lublin, Lublin, Poland; 3 Institute of Sociology, Faculty of Social Sciences, John Paul II Catholic University, Lublin, Poland; 4 Department of Medical Sciences, University of Udine, Udine, Italy; University of Auckland, NEW ZEALAND

## Abstract

**Background:**

Spirituality is vital in the holistic approach to nursing care. The personal spirituality of nurses has been documented to have an impact on the spiritual nursing care they provide.

**Aim:**

To validate the Polish version of the Spiritual Attitude and Involvement List (SAIL) among nurses and to describe spiritual attitudes and involvement of nurses as measured with the tool.

**Design:**

A cross-sectional, validation study was performed according to the STrengthening the Reporting of OBservational studies in Epidemiology guidelines.

**Methods:**

Study involving 163 nurses, with the use of four tools: (a) the SAIL; (b) the Self-Description Questionnaire; (c) the Scale of Spiritual Transcendence; and (d) the Brief Religious Coping questionnaire.

**Results:**

The Explanatory Factor Analysis identified six factors and 25 items were retained explaining a total variance of 67.96%. In the Confirmative Factor Analysis, acceptable index fit values were obtained. Correlations were found between SAIL and the Brief Religious Coping questionnaire, the Self-Description Questionnaire, and the Spiritual Transcendence Scale. Nurses reported the highest scores on ‘Meaningfulness’ (= 4.95 out of 6.0, Standard Deviation [SD] 0.56) and the lowest on the ‘Transcendent Experiences’ factor (= 3.21 out of 6.0, SD 0.93). A strong correlation was found between ‘Spiritual Activities’ and the religious involvement of nurses (*r =* 0.506), and between ‘Connectedness with nature’ and the nurses’ age and work experience (*r =* 0.315 and *r =* 0.321, respectively).

**Conclusion:**

The Polish version of SAIL shows acceptable psychometric properties and construct validity. With the validation of SAIL, future studies can be undertaken with the aim of measuring the personal spirituality of nurses in various settings (hospital vs. community), cultures, and countries, thus increasing the opportunity to compare findings. Moreover, more studies should be performed in order to assess whether there is any connection between personal spirituality and the spiritual nursing care provided to patients.

## Introduction

As spirituality is considered an essential part of the human condition, spiritual care must be seen as a vital component of holistic care of the patient and an important factor influencing quality of care [1]. The spiritual dimension of human beings has always been highlighted in nursing practice as a key element, from the early days of nursing conceptualisation by Florence Nightingale up to the current philosophy of humanistic nursing care by Jean Watson [2, 3]. According to her theory, care and caring requires from nurses personal, social, moral, and spiritual engagement [4].

When performing a non-systematic review of the literature using two key words: ‘spirituality’ and ‘nursing’ it is evident that a growing number of studies has been published in the last decades on different aspects of spirituality in the nursing discipline. The analysed aspects included the nurses’ personal spirituality and spiritual care [2, 3, 5, 6], the nurses’ spiritual competences and development [7–9], as well as the spiritual needs and outcomes of spiritual care interventions at the patient level [10–12].

Specifically, in their concept analysis, Ramezani and colleagues [6] defined seven main attributes of spiritual care as: the healing presence, the therapeutic use of self, the intuitive sense, the exploration of the spiritual perspective, the patient-centredness, the meaning-centred therapeutic intervention, and the creation of a spiritually nurturing environment. The literature suggests that a nurse’s personal spirituality is an antecedent of spiritual care and is associated with the nurse’s attitudes to provision of care. Activities as, for example, attending religious services, religious insights, different spiritual experiences, and personal values have been reported as impacting the sensitivity to patients’ spiritual needs and nurses’ interventions when these needs are fulfilled [6]. Additionally, being spiritually self-aware has been documented as increasing the capacity to provide spiritual care both in frequency and quality [9, 13–16].

Evaluating personal spirituality can help nurses and nursing students to self-reflect and promote growth, which in turn may positively influence the nursing care provided to patients [13, 17]. Moreover, evaluating personal spirituality with validated instruments might promote research studies in the field with international approaches. Therefore, with the aim of improving the quality of the tools available in the field, by considering the cultural implications of spirituality as well as by describing the spiritual attitude and involvement of nurses, a validation process of the Spiritual Attitude and Involvement List (SAIL) in the nursing field has been performed.

## Background

Several studies have documented that nurses address the patient’s spiritual needs when providing nursing care. However, this is done intuitively and with a variable frequency, even when they believe this is an important element of their practice [15, 18]. Moreover, nurses are also uncertain about what spirituality is and how it should be focused on while caring [15]. Professional and scientific debates regarding the need for regular assessments of spirituality as a standard of care as well as the meaning of spiritual nursing care have highlighted different approaches to the concept of spirituality: the way in which spirituality is conceptualised, embodied, and measured is often influenced by culture and religion [3, 19].

Spirituality is a complex phenomenon with different definitions and conceptualisations developed by scholars and a variety of perspectives can be found: a religious one with spirituality conceived as a connection to God; an existential one with spirituality as a meaning of life and a source of hope and strength; and also connectedness with oneself/others/nature [8, 14]. Reinert and Koenig [3] in their trend analysis on spirituality have reported that there are three ways in which spirituality has been defined to date, as (a) definitions underlying a strong connection with religion, (b) definitions underlying the lack of connection of spirituality with religion, and (c) definitions indicating that spirituality might or might not have a connection with religion. This approach is visible in the definition accepted by the European Association for Palliative Care (EAPC), in which three dimensions of spirituality have been included: existential challenges, value-based considerations and attitudes, and religious considerations and foundations [20]. In other words, religion may be considered as a specific form of spirituality, while spirituality may or may not be related to religion [12]. Separating spirituality from religion, however, is causing controversy among some scholars. In Clarke’s opinion [21], the nurses’ irresistible desire to describe spirituality neglecting the knowledge from other disciplines, resulting in a variety of definitions which are too subjective, ample or existential, has increased the complexity of its measurements, education, and its practical implementation. Furthermore, defining spirituality in nursing should be based on the analysis of conceptual and empirical literature, and according to the analysis made by Weathers and colleagues [22] there are three defining attributes of spirituality: connectedness, transcendence, and meaning in life.

Many scholars and professional international bodies, such as the World Health Organization, the International Council of Nurses and several nursing organisations at the national level, have highlighted the importance of spiritual care in daily practice [10, 14]. However, given the complexity of spirituality both conceptually and in its measurement [9], it is difficult to establish a gold standard tool to measure nursing spiritual care [23]. Despite this, a wide range of methods and instruments has been proposed to facilitate spiritual assessment with four main approaches: generic, quantitative, qualitative, and domain-based [10]. In an available systematic review [24] on instruments measuring spirituality in clinical research, a total of 35 tools have been identified: these have been developed with the purpose of assessing multiple dimensions of spirituality, classified as measures of general spirituality, spiritual well-being, spiritual coping, and spiritual needs. In a study by van Leeuwen and colleagues [12], a total of 120 tools were identified for spiritual assessment, demonstrating that the taxonomy of the concept of spirituality is very rich and there are many ways to describe spirituality indicators.

By accepting the definition of spirituality as given by the EAPC [20], our aim was to assess the validity of a tool measuring personal spirituality with the following characteristics: (a) useful for a heterogeneous group of individuals, as non-believers or followers of different religions; (b) including in the measurement’s structure the existential perspectives, reflections and attitudes based on universal values, as reflected in the nursing practice, (c) capable of addressing, if present, religious attitudes, and (d) already used in the clinical context. Several tools have been developed and validated to date measuring personal spirituality as reported in the review performed by Meezenbroek et al. [25] in which the main focus was on tools suitable for religious and non-religious individuals, e.g. Prague Spirituality Questionnaire [26], Spiritual Transcendence Scale [27] and Spiritual Well-Being Questionnaire [28]. However, according to their psychometric properties, the quality of instruments available has been documented as low [25] with the exception of the Spiritual Attitude and Involvement List (SAIL) that has reported good psychometric properties [29] and has been proposed for the assessment of personal spirituality in the clinical context [29].

The SAIL tool is based on spirituality defined as “*one’s striving for and experience of connection with the essence of life*” in three main dimensions: connectedness with oneself, connectedness with others and nature, and connectedness with the transcendent [29] and adopts a spirituality non-theistic approach, thus capable of measuring spiritual experiences among individuals belonging to different religions and with different secular backgrounds. Moreover, the SAIL has already been used in the clinical context firstly among patients to investigate overlaps between aspects of spirituality and well-being, to further clarify the measurement of spirituality [30], and to investigate the association of spirituality with negative emotions [31]; secondly, among nurses and nursing students [9, 13, 14, 32], mainly with the purpose of analysing their personal spirituality and its correlations with competences in spiritual nursing care.

The SAIL has not been widely used in religious populations, with some exceptions, as that documented among Muslim patients [31]. Since the Polish society is mostly Catholic [33], we have decided to consider this tool to measure its universality.

## Method

### Aim

The aim of this study is threefold: (a) to validate the Polish version of the SAIL among nurses, (b) to explore the construct validity of the SAIL by examining correlations, if any, with other instruments measuring spirituality; and (c) to describe spiritual attitudes and the involvement of nurses.

### Design

Cross-sectional, validation study design performed in 2018, following the STrengthening the Reporting of OBservational studies in Epidemiology guidelines (STROBE) [34].

### Participants

A convenience sampling method was used. Professionally active nurses who at the time of the study (a) were participants of postgraduate courses in one voivodship of eastern part of Poland; (b) were not attending modules developing spirituality in nursing both in the postgraduate course and in their life, and (c) were willing to participate, were included.

In the first phase (*test*) 200 participants were invited and 163 (81.5%) agreed to participate. In the second phase (*retest*) 163 participants were invited from the same group of nurses after three weeks and 120 (73.6%) agreed to participate.

### Instruments

The following instruments were used:

- the SAIL [29] tool divided into seven subscales with 26 items, reflecting three dimensions: (1) ‘Connectedness with oneself’ (subscales: Meaningfulness– 3 items; Trust– 4 items; Acceptance– 4 items); (2) ‘Connectedness with others and nature’ (subscales: Caring for others– 4 items, Connectedness with nature– 2 items); and (3) ‘Connectedness to the transcendent’ (subscales: Transcendent experiences– 5 items; Spiritual activities– 4 items). The tool has been validated in different populations: students, healthy individuals, adults interested in psychology, philosophy or spirituality, patients with curable cancer and patients with palliative cancer. Factorial, convergent and discriminant validity have been documented [29] with an internal consistency (Cronbach’s alphas (α)) ranging from 0.74 to 0.88 across sub-scales [13]. The metrics of the tool is based on a Likert-type scale scoring from 1 –the lowest, to 6 –the highest personal spirituality; therefore, a high score in the whole scale (>4) indicates a high level of spiritual attitude and involvement [14]. Permission for the use of the SAIL was obtained from the authors (correspondence available from authors).
- three additional instruments in order to discover any correlations between the SAIL scores and other tools measuring personal spirituality and religiosity as a method to check the SAIL’s construct validity [35]. When choosing tools, the following criteria were taken into account: their availability in the Polish language, their good psychometric properties for the Polish population, and their scope. On permission, the following instruments were used (correspondence available from authors):
  - the Self-Description Questionnaire [36] measuring spirituality and consisting of 20 items divided into three subscales: ‘Religious attitudes’ (7 items, α = 0.90), ‘Ethical sensitivity’ (7 items, α = 0.82), and ‘Harmony’ (6 items, α = 0.81). The scale was developed and validated in Poland;
  - the Scale of Spiritual Transcendence [37] developed and validated in Poland, measuring spirituality with two subscales: ‘Transcendence Proper’, and ‘Spiritual Openness’ with 11 items/each. Both subscales and the whole scale have been documented as being highly reliable, internally consistent (α = 0.89 and α = 0.80, respectively; for a total of α = 0.87) and stable over time;
  - the Brief Religious Coping (RCOPE) questionnaire [38] in its Polish adaptation [39] consisting of 14 items and measuring two strategies of religious coping: positive (7 items, α = 0.86) and negative (7 items, α = 0.74).

Moreover, some sociodemographic data (age, gender, level of education, work experience, religious beliefs) were also collected.

### Data collection

After gaining consent from the person responsible for the centres of postgraduate education for nurses and midwifes in the district of Lublin voivodship, paper and pencil copies of questionnaires were distributed among eligible nurses. The researcher explained the aims of the study and the procedure of data collection in two phases, firstly for the *test* phase, and secondly for the *retest* phase. Those who verbally consented to take part in the study were given the questionnaire. At the beginning of the questionnaire, a specific statement was reported specifying that filling in the questionnaire was understood as consent to take part in the study.

Nurses were asked to fill in the questionnaire after a lecture and leave it in a box placed in the secretary’s office. The respondents were informed that they could withdraw from the study at any time and return a void questionnaire. No pressure was applied on respondents; they were left free to ask questions and to obtain explanations.

### The SAIL cultural adaptation procedure

The translation of the English version of the SAIL tool into Polish was carried out in accordance with the guidelines of the International Test Commission [40]. Two English linguists translated the items from English to Polish, thus preparing an initial version of the Polish version of the tool. This version was the basis for the discussion of three specialists in spirituality: a psychologist, a theologist and a nurse working in this field. After their analysis, the draft version of the Polish version of the SAIL tool [29] was prepared and given to another English linguist for back-translation into English. Based on the translator’s comments, the first Polish version of SAIL was drafted. A pilot phase was carried out with the participation of 15 nurses to explore its feasibility and understandability: specifically, items 4 and 25 were highlighted as ambiguous. To establish their real meaning, the authors of the scale were contacted, after which the items were re-worded and included in the final version of the tool.

### Data analysis

The analyses were performed using the IBM SPSS Statistics 25 and IBM SPSS AMOS 25 software packages. The analysis was performed according to the study aims, as follows:

- with regards to the first aim, the factorial structure of the Polish version of the SAIL scale was assessed with the exploratory factor analysis (EFA) based on the principal component method and the Oblimin rotation for the degree of skewness delta = 0 (non-orthogonality of factors was assumed). Then, the confirmatory factor analysis (CFA) was performed by establishing the Comparative Fit Index (CFI, > 0.95 as excellent), the Tucker-Lewis Index (TLI, > 0.95 as excellent), the Root Mean Square Error of Approximation (RMSEA, < 0.06 as good), and the Standardised Root Mean Square Residuals (SRMR, < 0.08 as good fit). The test of close fit (PCLOSE, ≥0.05; the value of PCLOSE associated with RMSEA should be greater than .05 to ensure a close fit) was also evaluated. The adjusted goodness-of-fit index (AGFI >0.8 as acceptable fit level) and the goodness-of-fit index (GFI>0.9 as satisfactory fit) was calculated [41]. Next, factors were labelled in accordance with the original version of the tool [29]; the analysis of the contents of the items was first assessed independently and then jointly by three experts–two nurses interested in spirituality and one sociologist of theology working on this topic. Furthermore, in order to assess the reliability of the scale, the internal consistency and the repeated measurement method (test-retest) were performed. Specifically, the aim of the retest was to measure the stability of the tool and to find out to what extent the test results that concerned spirituality are independent of random factors associated with the subject as, for example, the negative personal experiences or the organization of data collection.
- with regards to the second aim, the construct validity was estimated on the basis of the correlations (Pearson, *r*) of the Polish version of SAIL factors and (i) the Brief RCOPE questionnaire, (ii) the Self-Description Questionnaire, and (iii) the Spiritual Transcendence Scale.
- with regards to the third aim, a descriptive statistic was used to calculate frequencies, percentages, averages and Standard Deviations (SD) or Confidence of Intervals (CI, 95%) as well as the skewness and kurtosis of distributions. Correlations, if any, were explored with the Pearson (*r*) or the Spearman (rho) test, according to the nature of the variables under study.

### Ethical issues

The research protocol of this study was accepted by the Ethical Committee at the accessible Medical University (No. KE-0254/128/2018). Nurses were invited to take part in the study on a voluntary basis. All of them were informed about the aims of the study and the entire procedure of data collection. Participants were assured that all data collected during the research process would be treated confidentially. Verbal consent was obtained from respondents–those who agreed to participate in a study were given the questionnaire. At the beginning of the questionnaire, the statement at the top reiterated that filling in the questionnaire was taken as consent to participate in the study. The decision to obtain the consent for participation in the study was undertaken in this way to protect respondents’ anonymity and privacy avoiding additional documents requiring their signature. They could withdraw from the study at any time by declining to fill in the questionnaire. During the data collection a researcher was available if any doubts or questions emerged.

## Results

### Participants

A total of 163 nurses participated in the first phase (*test*). Their average age was 36 years (SD = 10.2); at the time of the study they had been working as nurses for an average of 12 years (SD = 11.2). In the second phase (*retest*), 120 nurses participated from the previous group; their average age was 37 years (SD = 9.9) and they had been working as nurses for an average of 12 years (SD = 11.2) as reported in Table 1.

**Table 1:** Participants.

Variables		Test (n = 163)		Retest (n = 120)	
		n	%	n	%
Gender	Female	153	93.9	114	95.0
	Male	7	4.3	5	4.2
	Missing data	3	1.8	1	0.8
Education	Diploma in Nursing	16	9.8	10	8.3
	Bachelor degree in Nursing	92	56.4	72	60.0
	Master degree in Nursing	48	29.4	36	30.0
	Missing data	7	4.3	2	1.7
Employment	General Practitioner Practice	26	16.0	17	14.2
	Hospice/Long term care	20	12.3	12	10.0
	Medical hospital wards (e.g., internal medicine, geriatrics, dermatology, pulmonology)	14	8.6	14	11.7
	Surgical hospital wards (e.g., surgery, operating room, ICU)	51	31.3	44	36.7
	Mental health care	19	11.6	16	13.3
	Other	33	20.2	17	14.2
Place of residence	City	84	51.5	61	50.8
	Village	75	46.0	58	48.3
	Missing data	4	2.5	1	0.8
Religious declaration	Believing	142	87.1	108	90.0
	Catholic	132	93.0	102	94.5
	Orthodox	2	1.4	1	0.9
	Jehovah’s Witness	1	0.7	1	0.9
	Lack of answer	7	4.9	4	3.7
	Unbelieving	16	9.8	10	8.3
	Not reported	5	3.1	2	1.7

ICU–Intensive Care Unit.

### Exploratory factor analysis

The Exploratory Factor Analysis was performed and the validity of the choice of the factor analysis model was formally confirmed using the Kaiser-Meyer-Olkin index (KMO = 0.82) and the Bartlett sphericity test (χ^2^ = 1513.40, p <0.001). Initial analysis of the correlation matrix revealed the existence of numerous statistically significant correlations (from moderate and high intensity–up to *r* 0.76) between items. At the EFA, six factors were obtained, one less than in the original version [29], which explained 67.96% of the total variance as reported in Table 2.

**Table 2:** Polish version of the SAIL questionnaire: Explorative Factor Analysis and internal consistency (= 163).

Factors, item loadings						
SAIL items (*)	1	2	3	4	5	6
	Transcendent experiences	Spiritual activities	Connectedness with nature	Meaningfulness	Acceptance	Trust
Item 11	0.432					
Item 23	0.491					
Item 21	0.613					
Item 19	0.649					
Item 20	0.680					
Item 25	0.701					
**Variance %**	**24.891**					
**α**	**0.812**					
Item 4		0.421				
Item 10		0.652				
Item 26		0.654				
Item 22		0.684				
Item 24		0.718				
**Variance %**		**9.847**				
**α**		**0.757**				
Item 14			0.842			
Item 5			0.868			
**Variance %**			**7.566**			
**α**			**0.798**			
Item 2				0.401		
Item 12				0.541		
Item 17				0.599		
Item 16				0.687		
Item 18				0.834		
**Variance %**				**6.350**		
**α**				**0.748**		
Item 13					0.403	
Item 15					0.570	
Item 9					0.647	
Item 3					0.800	
**Variance %**					**4.693**	
α					**0.627**	
Item 1						0.578
Item 8						0.813
Item 6						0.870
**Variance %**						**4.614**
**α**						**0.706**
**Total Variance %**						**67.96**

SAIL**—**the Spiritual Attitude and Involvement List; α - Cronbach Alpha

(*) item numbers are coming from original version of the SAIL, which is available at: https://www.hdi.nl/0.9.2/wp-content/uploads/2014/10/SAIL-questionnaire-English.pdf.

The validation process of the scale was published in: Meezenbroek E, Garssen B, van den Berg M, Tuytel G, van Dierendonck D, Visser A, Schaufeli W. Measuring spirituality as a universal human experience: development of the Spiritual Attitude and Involvement List (SAIL). J Psychos Oncol. 2012; 30: 141–167. https://doi.org/10.1080/07347332.2011.651258.

The matrix model was analysed, including each of the factor’s items with a factor load value of >0.40. Additionally, on the basis of the graphic criterion of eigenvalues (scree plot), it was found that the 6-factor solution was the most adequate in the Polish SAIL version. According to the loads, the item number 7 from the original scale (‘*I am receptive to other people’s suffering’*) did not reach the criterion value as the load value was <0.40. For this reason, the item has been excluded from the final model.

The factors were labelled as follows: (1) ‘Transcendent experiences’, (2) ‘Spiritual activities’, (3) ‘Connectedness with nature’, (4) ‘Meaningfulness’, (5) ‘Acceptance’, and (6) ‘Trust’, as reported in Table 2. Analysis of correlations between the SAIL factors indicates that they are significantly positively correlated (p<0.05), predominantly to a moderate extent (from *r =* 0.179 to = 0.538).

### Confirmatory factor analysis

To check whether a 6-factor solution was well suited to the data, a CFA was performed. While the chi-square test results turned out to be statistically significant (χ^2^ = 382.02, p <0.001), it should be noted that they strongly depend on the sample size, the number of variables, the number of free parameters or deviations from the normality of the distribution. Other adjustment measures with acceptable values were referenced, indicating a good adjustment of empirical data to the model: RMSEA = 0.054; RMSEA- LO = 0.042; RMSEA- LO = 0.065; Pclose = 0.286; AGFI = 0.807; CFI = 0.910; SRMR = 0.075. A slightly lower value than the acceptable one was obtained for the GFI index (= 0.846).

### Reliability

The Cronbach’s alpha internal consistency was α 0.711 (95% CI = 0.636 = 0.775) as reported in Table 2. The factor reporting the highest α value was ‘Transcendent experiences’ (= 0.812) while the lowest was ‘Acceptance’ (= 0.627).

### Stability

After 3 weeks (*retest*), the tool was re-administered to establish its stability over time. As reported in Table 3, the Pearson correlation coefficients for each factor reported statistically significant findings from *r* = 0.790 (‘Connectedness with nature’) as the highest correlation, to *r* = 0.623 (‘Trust’) as the lowest correlation.

**Table 3:** Test-Retest: Stability results for Polish SAIL subscales (= 120).

Factors	(1)	(2)	(3)	(4)	(5)	(6)
Transcendent experiences (1)	0.733^**^	-	-	-	-	-
Spiritual activities (2)	-	0.787^**^	-	-	-	-
Connectedness with nature (3)	-	-	0.790^**^	-	-	-
Meaningfulness (4)	-	-	-	0.759^**^	-	-
Acceptance (5)	-	-	-	-	0.759^**^	-
Trust (6)	-	-	-	-	-	0.623^**^

SAIL**—**the Spiritual Attitude and Involvement List

** p<0.001.

### Construct validity

As reported in Table 4, the SAIL factors showed positive correlations with the overall score and with almost all subscales of the Self-Description Questionnaire. Correlations were statistically significant positive, mostly moderately strong (from *r* = 0.225 to *r* = 0.703); the ‘Acceptance’ and ‘Trust’ factors of the SAIL were in turn not correlated with the ‘Ethical sensitivity’ subscale (*r* = 0.140; *r* = 0.127).

The SAIL factors showed also statistically significant positive correlations with the overall score of the Spiritual Transcendence Scale from *r* = 0.218 to *r* = 0.714 with the exception of the ‘Acceptance’ factors and that ‘Transcendence proper’ (Table 4).

**Table 4:** Correlations of the SAIL with the Self-Description Questionnaire, the Spiritual Transcendence Scale, and the Brief RCOPE questionnaire (= 144).

	The Self-Description Questionnaire				The Spiritual Transcendence Scale			The Brief RCOPE questionnaire	
SAIL factors	General results	Religious attitudes	Ethical sensitivity	Harmony	Transcendence proper	Spiritual openess	The Spiritual transcendence	Positive strategies	Negative strategies
	(^1^)	(^1^)	(^1^)	(^1^)	(^1^)	(^1^)	(^1^)	(^1^)	(^1^)
Transcendent experiences	0.489**	0.651**	0.377**	0.282**	0.555**	0.286*	0.520**	0.479**	0.382**
Spiritual activities	0.703**	0.528**	0.459**	0.355**	0.714**	0.286*	0.632**	0.758**	-0.212*
Connectedness with nature	0.369**	0.671**	0.296**	0.225**	0.367**	0.264*	0.378**	0.332**	0.137
Meaningfulness	0.405**	0.409**	0.467**	0.363**	0.277*	0.284*	0.324**	0.222*	0.107
Acceptance	0.260*	0.438**	0.140	0.268**	0.151	0.301**	0.243**	0.102	-0.109
Trust	0.307**	0.675**	0.127	0.334**	0.218*	0.278*	0.280*	0.125	-0.127

(^1^) *r* Pearson

** p<0.001

*p<0.01.

SAIL—the Spiritual Attitude and Involvement List.

Correlation of SAIL factors with that of the Brief RCOPE questionnaire showed a significant positive correlation between the ‘Transcendent experiences’ and positive and negative religious strategies for coping with stress (*r* = 0.479 and *r* = 0.382, respectively). Furthermore, the ‘Spiritual activity’ factor correlated positively with positive (*r* = 0.758) and negatively with negative coping strategies (*r* = -0.212). A correlation also emerged between the ‘Connectedness with nature’ (*r* = 0.332) and the ‘Meaningfulness’ (*r* = 0.222) subscales, and positive stress coping strategies (Table 4).

### Participants’ spirituality according to the Polish version of SAIL

Our participants reported the highest scores on the ‘Meaningfulness’ (= 4.95, SD 0.56) while the lowest on the ‘Transcendent experiences’ factor (= 3.21, SD 0.93), as presented in Table 5.

**Table 5:** Polish version of SAIL: Descriptive statistics and correlations (n = 163).

SAIL factors^§^	M	SD	A	K	Min-Max	Age^(^^1^^)^	Work experience years ^(^^1^^)^	Religious involvement (yes vs. no) ^(^^1^^)^
Transcendent Experiences	3.21	0.93	0.40	0.02	1.20–6.00	0.134	0.151	0.264*
Spiritual activities	3.95	0.90	-0.02	-0.05	1.40–6.00	0.258*	0.238*	0.506**
Connectedness with Nature	4.81	1.00	-0.72	0.13	1.50–6.00	0.315**	0.321**	0.188**
Meaningfulness	4.95	0.56	-0.35	0.15	3.20–6.00	-0.057	-0.114	0.146
Acceptance	3.46	0.51	-0.56	0.70	1.80–4.60	0.069	0.038	0.177*
Trust	4.09	0.80	-0.34	0.47	2.00–6.00	0.221*	0.133	0.079

^**§**^Likert:1-not at all, 2-hardly at all, 3-samewhat, 4-to reasonable degree, 5-to high degree, 6-to a very high degree.

(^1^)*r* Pearson

(^2^) rho Spearman

**p<0.001

*p<0.01.

A -skewness; K -kurtosis; M -mean; SD -standard deviation; SAIL—the Spiritual Attitude and Involvement List.

Table 5 also shows that skewness was mainly negative among factors with the exception of ‘Transcendent experience’ which equalled = 0.40; for what concerns kurtosis, these were mainly positive close to 0, with the exception of the ‘Spiritual activities’ factor which was negative (= - 0.05).

Age significantly correlated with the ‘Spiritual activities’ (*r* = 0.258, p<0.01), ‘Connectedness with nature’ (*r* = 0.315; p<0.001) and ‘Trust’ (*r* = 0.221, p<0.01) factors. Similarly, years of work experience significantly correlated with ‘Spiritual activities’ (*r* = 0.238, p<0.01) and ‘Connectedness with nature’ (*r* = 0.321; p<0.001), as reported in Table 5. With regards to religious involvement, it was moderately correlated with the ‘Spiritual activities’ factor (rho = 0.506; p<0.001) followed by ‘Transcended experience’ (rho = 0.264; p<0.01), as reported in Table 5.

## Discussion

The aim of this study was to assess the psychometric properties of the SAIL in the Polish context and to measure spiritual attitude and involvement among nurses. Several tools measuring personal spirituality are available in Poland as, for example, the Self-Description Questionnaire [36]; the Spiritual Sphere Questionnaire [42]; and the Scale of Spiritual Transcendence [37]. However, they are used mostly by psychologist or sociologist of religion [39] and have not been used in the nursing context. To our best knowledge, no research has been performed to date regarding personal spirituality of nurses by using a validated questionnaire in Poland. Therefore, the adaptation and the validation of the SAIL to the Polish culture, as well as testing its psychometric properties among nurses, is of great importance for future research both at the national and at the international levels.

### Findings of the validation process

The Polish version of SAIL shows satisfactory psychometric properties. First, the EFA of the Polish version did not allow the reconstruction of its original 7-factor structure: in fact, a 6-factor structure has emerged explaining the 67.96% of the total variance, similar to that of the original version of the SAIL tool (= 68%) [29]. Moreover, the 6-factor structure was confirmed by the CFA with acceptable indices of fit.

According to the findings, only one factor has remained unchanged–‘Connectedness with Nature’; furthermore, in the ‘Transcendent Experiences’ and ‘Spiritual Activities’ subscales one item was added (number 11 and 4, respectively). In addition, as compared to the original version, items composing the ‘Meaningfulness’ and ‘Caring for others’ subscales collapsed in a unique factor labelled ‘Meaningfulness’. The findings also highlighted two factors that were not entirely consistent with those identified in the original SAIL: these were labelled ‘Trust’ and ‘Acceptance’, respectively, as in the original version of the tool and they even consisted of different items. Therefore, our findings influenced three dimensions identified in the original tool [29] as: (1) ‘Connectedness to oneself’, (2) ‘Connectedness to others and nature’, and (3) ‘Connectedness to the transcendence’.

Some issues with the ‘Trust’ SAIL factor have been reported by Visser and colleagues [30], who performed a study to identify overlaps between spirituality and well-being. This factor strongly correlated with the three aspects of well-being (‘Vitality’, ‘Peace’, and ‘Pleasure’) measured by one subscale of Health and Diseases Inventories. Therefore, experience of trust as the ability to cope with difficulties in life has been reported as being not unique to spirituality: according to this finding, authors suggest that this factor should not be included in instruments measuring spirituality [30]. However, given that in our research the ‘Trust’ factor was composed of other items, different from those included in the original SAIL version [29], we have decided to include it.

Moreover, according to the EFA, the subscale ‘Caring for others’ was excluded from the final version of the tool by moving three of its items to the subscale ‘Meaningfulness’, and by totally deleting one as its load value was < 0.40. The deleted item number 7 from the original scale (‘*I am receptive to other people’s suffering’)* reflects a prerequisite and an essential element of the fundamental care of the nursing profession and not a personal attribute. Therefore, being widely recognised as an essential component, as also emerged in the averages obtained by the surveyed nurses (>5.00 –data available from authors), the item has been removed.

In view of the above, changes which occurred in the factorial model of the Polish version of SAIL suggest that spirituality is culturally sensitive and exhibits the need for continuous research in culturally different contexts.

The internal consistency of the Polish version was α = 0.711, while the test-retest reliability ranged from 0.627 to 0.812, thus reflecting good properties. Authors have established the value of 0.7 as Cronbach’s acceptable alpha value [43] and values < 0.6 not acceptable [44–46]. However, values from 0.60 to 0.70 have been suggested as acceptable in exploratory studies, while in advanced stages of research values from 0.70 to 0.90 can be considered satisfactory. The internal consistency of the SAIL original version ranged from 0.74 to 0.88 [13] while in a study including cancer patients it ranged from 0.75 to 0.89 and its 6-month test-retest reliability ranged from 0.64 to 0.72 [30].

By using external tools represented by three instruments available in Polish, positive correlations were found between the SAIL version and the majority of subscales of these instruments, thus proving the construct validity of the SAIL in its Polish version. Specifically, all SAIL factors were positively correlated with the ‘Religious attitudes’ and ‘Harmony’ subscales and, to a lesser extent, with the ‘Ethical sensitivity’ of The Self-Description Questionnaire. Similar findings emerged for all subscales of The Spiritual Transcendence Scale. At the same time, some positive correlations emerged between ‘Transcendent Experiences’, ‘Spiritual Activity’, ‘Connectedness with Nature’ and ‘Meaningfulness’ and positive stress coping strategies as measured with The Brief RCOPE questionnaire. This seems to demonstrate that personal spirituality in these four subscales of SAIL is related to the religious coping of difficult situations and that, in general, spirituality and religiosity are positively connected in our respondents.

### Participants’ spirituality according to the Polish version of SAIL

Average results obtained by nurses in the Polish version of SAIL ranged from 3.21 in the ‘Transcendent Experiences’ subscale to 4.95 in the ‘Meaningfulness’ subscale. In the SAIL 8-subcale version, average values have been reported as lower and oscillating, e.g. from 2.3 (‘Transcendent experiences’) to 4.4 (‘Caring for others’) among students [29]. Other comparisons are limited with the Polish findings due to the different factorial structure of the tool. However, in a study by Visser and colleagues [30] in cancer patients, the highest-scored factors in a 7-subscale were ‘Connectedness with nature’ (= 4.73) and ‘Caring for others’ (= 4.69) while the lowest was ‘Transcendent experiences’ (= 2.44) and ‘Spiritual activities’ (= 3.00). Moreover, in a longitudinal study by Ross and colleagues [9] involving nursing students at the start of their education the lowest score was reported in the ‘Connection to the transcendent’ containing the ‘Transcendent Experiences’ and the ‘Spiritual Activities’ subscales (= 2.9), while the highest in the ‘Connection to others’ including the ‘Caring for others’ and the ‘Connectedness with nature’ subscales (= 4.6). These values did not change at the end of nursing education; however, students reported slightly higher results in each of three dimensions (‘Connectedness to oneself’, ‘Connectedness to others and nature’, and ‘Connectedness to the transcendent’) of the SAIL (from 3.0 to 4.8), thus reflecting their personal spiritual development [9]. A similar tendency was revealed in a study by van Leeuwen and Schep-Akkerman [13] involving professionally active nurses from three different environments: hospital, mental health, and care homes. They reported low scores in ‘Connectedness to the transcendent’ (= 2.9 for hospital nurses, 3.0 for mental health nurses, and 3.3 for home care nurses) and high in ‘Connectedness to others and nature’ (= 4.4 for hospital nurses, 4.7 for mental health nurses, and 5.0 for home care nurses) [13].

As for the demographic variables describing the surveyed nurses, a strong statistical correlation was found between ‘Spiritual Activities’ and religious beliefs of nurses, and between ‘Connectedness with nature’ and nurses’ age and work experience. Similarly, van Leeuwen and Schep-Akkerman [13] recorded that age and religious beliefs of nurses correlated with all three dimensions of the SAIL, thus suggesting that these dimensions of spirituality increase with life experiences as well as with religiosity. This, again, serves to show that in our study personal spirituality is influenced by the religiousness of the surveyed nurses and, additionally, by their growing experience as a person and as a nurse. According to van Leeuwen and co-authors [47], older and experienced nurses are self-assessing themselves as being more competent in providing spiritual care.

### Study limitations

This study has several limitations. We have approached a convenience sample of nurses by involving a limited number of them working in the same region of Poland and in a religiously and culturally homogenous community with the majority of nurses describing themselves as Catholic. Even though it was our aim to check the psychometric properties of the SAIL in the group of religious respondents, the study would benefit from comparison with those with different life views. Furthermore, in establishing the sample size, we have considered the ratio of 1 item of the tool per 5 participants [48, 49]. There is no clear agreement regarding the minimum of sample subjects when validating tools [50] however, the ratio 1:10 is recommended [51]. Therefore, further cumulative evidence both on the tool validity and the findings is suggested. Some demographic data has been collected among nurses, therefore limiting further analysis on individual factors influencing spirituality.

## Conclusions

This is the first research in Poland measuring the personal spirituality of nurses with the use of the SAIL tool. The Polish version of SAIL resulted in a 6-factor model including 25 items, thus with a different structure from the original version impacting the three-dimension concept on which the original SAIL was developed. The changes required in the factorial structure suggest that spirituality is culturally sensitive and exhibits the need for continuous research in culturally different contexts. Moreover, the Polish version of SAIL shows acceptable psychometric properties and construct validity.

According to the findings, low scores in spiritual attitude and involvement participants nurses reported in ‘Transcendent Experiences’, ‘Acceptance’, and in ‘Spiritual activities’. On the other side, high scores have emerged in the ‘Connectedness with nature’, ‘Meaningfulness’, and ‘Trust’ subscales. With the Polish validation of the SAIL, future studies can be undertaken with the intent to measure personal spirituality of nurses in diverse settings (hospital vs. community), cultures, and countries, thus increasing the opportunity to compare findings. Moreover, with a valid scale available, more studies should be performed in other to assess whether there is any connection between personal spirituality and the spiritual nursing care provided to patients, adding value to existing studies using SAIL in culturally heterogenous populations.

## Supporting information

S1 Checklist STROBE Statement—Checklist of items that should be included in reports of *cross-sectional studies*. (DOCX)
